# L’enclouage centromédullaire dans les fractures complexes de l’extrémité supérieure de l’humérus: résultats préliminaire à propos de 6 cas

**DOI:** 10.11604/pamj.2016.25.54.9730

**Published:** 2016-09-30

**Authors:** Louaste Jamal, Taoufik Cherrad, Hicham Bousbaa, Mohammed Wahidi, Larbi Amhajji, Khalid Rachid

**Affiliations:** 1 Service de Chirurgie Orthopédique et Traumatologique, Hôpital Militaire Moulay Ismail BP 50000, Meknès, Faculté de Médecine et de Pharmacie de Fès, Maroc

**Keywords:** Fracture, humérus, ostéosynthèse, enclouage, prothèse inversée, Fracture, humerus, osteosynthesis, nailing, reverse prothesis

## Abstract

L’enclouage centromédullaire antérograde s’est imposé comme un des traitements de référence des fractures céphalotubérositaires à deux, trois et quatre fragments. Nous présentons une étude rétrospective de 06 patients ayant bénéficié d’un enclouage centromédullaire depuis janvier 2012. Le Recul moyen est de 12 mois, l’âge moyen était de 57 ans. Evaluation clinique se basait sur le score de Constant et Murley, en brut et pondéré en fonction de l’âge et du sexe, avec un comparatif avec le coté sain. Le bilan radiologique nous a permis d’évaluer la consolidation osseuse, l’apparition d’Ostéonécrose de la tête ou arthrose post traumatique. Surveille aussi l’Etat des tubérosités avec l’existence ou non d’ostéolyse du trochiter. Elle recherche aussi les critères de bonne réduction à savoir l’angle calotte céphalique et l’axe diaphysaire (αF) sur les clichés de face. Tous les patients ont été traités selon la même technique chirurgicale. Le score de Constant et Murley sur l’ensemble des patients était de 64.13 points. Le score pondéré en fonction de l’age et du sexe était de 73%. Les mobilités articulaires étaient en moyenne sur l’ensemble des patients 116° en élévation antérieure, 99.9° en élévation latérale, et 42° en rotation externe. Angle αF moyen s’élève à 42°. Tous les patients présentaient des critères de bonne réduction à savoir αF. L’enclouage centromédullaire permet une synthèse osseuse simple et au prix d’un abord limité avec des résultats fonctionnels très prometteurs. La comminution fracturaire et l’ostéoporose peuvent limiter ces indications.

## Abstract (en)

Antegrade intramedullary nailing has become the gold standard to treat two-, three-, and four-part cephalo-tuberosity fractures. We report a retrospective study of 6 patients who have undergone centro-medullary nailing since January 2012. Mean follow-up was 12 months; the average age was 57 years. Clinical evaluation was based on gross and weighted Constant and Murley score, based on the age and sex, compared to the “normal” body surface area. Radiological assessment allowed us to evaluate bone healing, the occurrence of osteonecrosis of femoral head or of post-traumatic arthrosis. Radiological examination focused even on the state of the tuberosities and on the existence or not of osteolysis of the greater tubercle of the humerus. It was also used to identify the criteria for good reduction, namely cephalic cap angle and diaphyseal axis (αF) in face views. All patients underwent the same surgical procedure. Constant and Murley score for all patients was 64.13 points. Weighted score based on age and sex was 73%. Joint mobility was estimated at an average of 116° for all patients in anterior elevation, 99.9° in lateral elevation, and 42° in external rotation. Average αF angle was 42°. All patients met criteria for good reduction, namely αF. Centro-medullary nailing allows simple and cost-effective bone synthesis with very promising functional results. Comminuted fractures and osteoporosis may limit these indications.

## Introduction

Les Fractures proximales de l’humérus sont très fréquentes représentant 5% de toutes les fractures. Leur répartition est bimodale touchant préférentiellement le sujet âgé ostéoporotique après un traumatisme à faible énergie ou plus rarement le sujet jeune par mécanisme à forte cinétique. Elles sont Caractérisées par les difficultés de leur traitement, l’absence de technique de référence et certaines controverses: lesquelles opérées? Quelle technique pratiquée: prothèse? Ostéosynthèse a ciel ouvert? Ou percutanée? L’enclouage centromédullaire antérograde de l’humérus s’impose comme un des traitements de référence des fractures a deux, trois et quatre fragments.

## Méthodes

**Figure 1:**
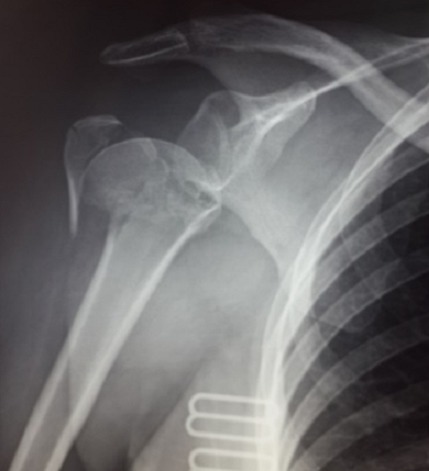
Contrôle préopératoire par amplificateur de brillance, noter la bonne réduction et le bon positionnement des vis céphaliques

**Figure 2:**
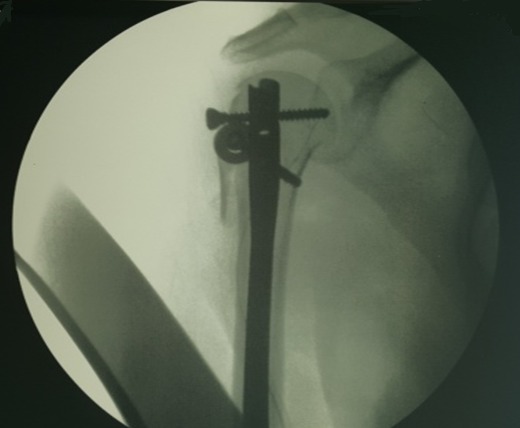
Femme âgée de 50 ans présentant une fracture a 3 fragments

**Figure 3:**
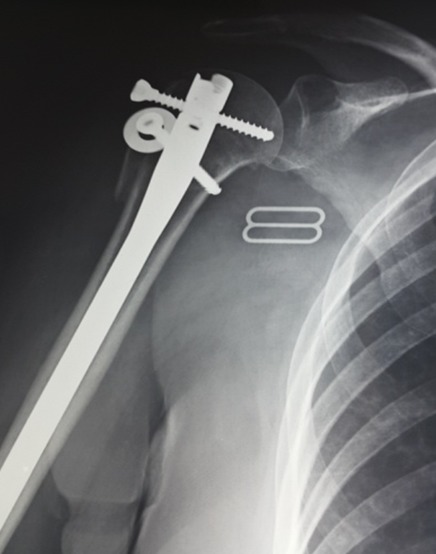
Contrôle radiologique a 3 mois d’évolution, noter la consolidation des fragments en bonne

Nous présentons une étude rétrospective de 06 patients ayant bénéficié d’un enclouage centromédullaire depuis janvier 2012. Le Recul moyen est de 12 mois avec des extrêmes de 08 mois et 24 mois. Cinq malades ont été opérés le même jour et un seul le lendemain. On avait 03 hommes et 03 femmes, l’âge moyen était de 57 ans (53 ans-61 ans). Le Coté dominant était atteint dans tous les cas (Tableau 1). L'évaluation clinique se basait sur le score de Constant et Murley [1], en brut et pondéré en fonction de l’âge et du sexe, avec un comparatif avec le coté sain. Le bilan radiologique nous a permis d’évaluer la consolidation osseuse, l’apparition d’Ostéonécrose de la tête ou arthrose post traumatique de surveiller aussi l’état des tubérosités avec l’existence ou non d’ostéolyse du trochiter. Elle recherche aussi les critères de bonne réduction à savoir l’angle calotte céphalique et l’axe diaphysaire (αF) sur les clichés de face (αF normal est de 45° (30°et 60°)) et la Présence d’une bascule antérieure ou postérieure. Tous les patients ont été traité selon la même technique chirurgical; en Position demi assise; sous AG. L’abord était supéro-latéral de 4 cm, avec incision de la coiffe sur 1 cm afin d’introduire le clou a la jonction trochiter cartilage. (Même plus en dedans en zone cartilagineuse pour les fractures déplacées) Plus le fragment articulaire est grand plus la réduction est plus aisée et meilleurs. Il ne faut jamais hésiter à enfouir le clou afin d’éviter tous conflits articulaire secondaire. La fixation des fragments se fait par 2 vis céphaliques au minimum dont on a le choix multiples de leur positionnement. Le montage était dynamique et jugée satisfaisant en peropératoire (Figure 1, Figure 2, Figure 3). Les patients sont sortis de l’hôpital avec bondage coude au corps pendant 06 semaines. La rééducation passive a été débutée à la 3^ème^ semaine pour décoaptation de la coiffe), le travail actif a été entrepris après la 6^ème^ semaine.

**Tableau 1:** Répartition de la série selon les différents types fracturaires

	Fr a 2 fragments	Fr a 3 fragments	Fr a 4 fragments	Total (06 cas)
Nombre de patients	1	2	3	6
Age moyen	55	57	59	57
Femmes	1	0	2	3
Hommes	0	2	1	3
Coté dominant	1	1	2	

## Résultats

**Figure 4:**
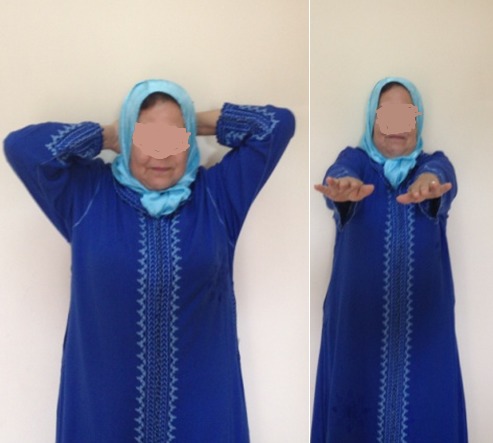
Très bon résultat fonctionnel a 3 mois postopératoire

**Les résultats fonctionnels:** (Figure 4) le score de Constant et Murley sur l’ensemble des patients était de 64.13 points. Le score pondéré en fonction de l’age et du sexe était de 73%. Les mobilités articulaires étaient en moyenne sur l’ensemble des patients 116° en élévation antérieure, 99.9° en élévation latérale, et 42° en rotation externe. Le score moyen de la douleur était de 11/15 (Tableau 2).

**Tableau 2:** Résultat fonctionnel selon le type fracturaire

	Neer 2	Neer 3	Neer 4	Total
C Brut	70.5	64.3	57.6	**64.13**
C Pondéré	84%	75.5%	67%	**73%**
Elévation ant	131°	122°	95°	**116°**
Elévation latérale	115°	105°	79°	**99.9°**
Rotation externe	51°	43°	31°	**42°**
Douleur	12.5	11	10	**11**

**Figure 5:**
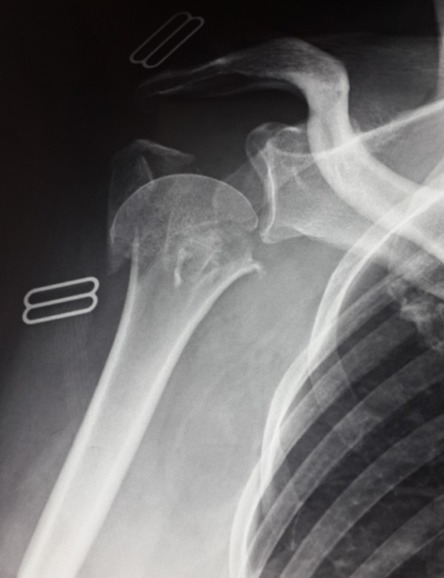
Fracture à 4 fragments chez une patiente âgée de 58

**Figure 6:**
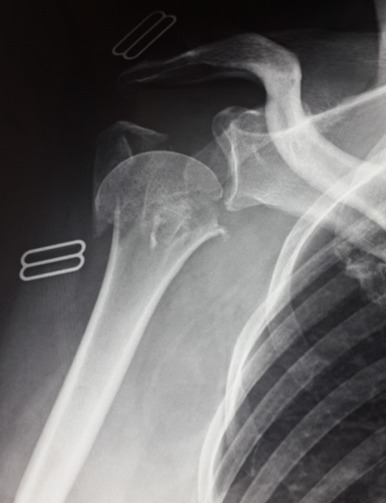
Contrôle radiologique à 4 mois évolution noté bien la consolidation avec une très bonne réduction

**Evaluation radiologique et corrélation radio clinique:** (Figure 5, Figure 6) angle αF moyen s’élève à 42°. Tous les patients présentaient des critères de bonne réduction à savoir αF (Tableau 3).

**Tableau 3:** Critères radiologiques de réduction chez nos patients en fonction de leur fracture

	Neer 2	Neer 3	Neer 4	Total
αF	47°	40.5°	39°	42.16°
Mauvaise réduction	0	0	0	0
Bascul post	0	0	0	

**Complications:** il n’a pas été mis en évidence de signes d’infection précoce ou tardive, pas de déplacement secondaire ou algodystrophie. Par ailleurs on n’a pas noté la survenue de pseudarthroses ou lyse du trochiter. Au dernier recule on n’a pas eu d’arthrose ou de nécrose.

## Discussion

Il reste clairement établi que les fractures peu déplacées plus comminutives peuvent satisfaire d’un traitement non chirurgical [2, 3]. Zeto [4] a montré des scores fonctionnel et clinique satisfaisants après traitement orthopédique des fractures multi-fragmentaires déplacées des patients âgés en moyenne de 70 ans. Mais c’est pour les fractures à 3 et 4 fragments que les indications diverges. Nombreuses sont les types d’ostéosynthèses: enclouage centromédullaire antérograde [5–7] ou rétrograde [7], plaque vissée [8, 9], embrochage fasciculé, hemiarthroplastie [10] ou prothèse totale inversée. L’enclouage centromédullaire offre un montage solide au prix d’un abord limité sur la coiffe [3]. Le clou permet la mise en place 4 vis céphalique fixant solidement les tubérosités. Il n’est pas toujours facile de les synthéser sous contrôle scopique en peropératoire. Chez le sujet jeune la plaque vissée peut être un choix judicieux, qui permet une meilleure fixation des tubérosités sous contrôle de la vue. Les critères de réduction radiologique ne sont pas corrélés à l’évolution arthrosique à long terme. L’angle aF doit être optimale entre 30° et 60°, seule garant d’une bonne réduction. L’état des tubérosités est un élément de pronostic majeur. Mais le risque d’Ostéonécrose de la tête reste plus important lié surtout à la manipulation directe des fragments et en fonction de la hauteur du trait fracturaire par rapport à l’axe vasculaire. Plus ce trait sépare un bec métaphysaire importante, plus le risque d’évolution vers l’ostéonécrose diminue [11]. Nous On n’a pas eu d’ostéonécrose, peut-être parce que on a beaucoup moins de patients que les autres séries qui avaient des taux variant de 3% à 43.7% [3, 8, 9, 12, 13]. Cependant ces taux montrent la difficulté de prédiction clinique dès la phase initiale. Cependant il existe un phénomène de revascularisation secondaire précoce de la tête humérale connu sous le nom de creeping substitution [14] semble venir diminuer ce taux d’ostéonécrose à distance. De plus il n y a pas une corrélation radio-clinique entre la sévérité des signes radiologiques de nécrose et les scores fonctionnels des patients [12, 13]. Donc il semble que malgré le risque important d’ostéonécrose, il faut effectuer en première intention une ostéosynthèse solide par plaque vissée ou clou centromédullaire en fonction de l’âge et la comminution fracturaire. Si la gêne fonctionnelle reste importante il y a toujours la possibilité de reprise chirurgicale par arthroplastie. D’autres auteurs font du risque d’ostéonécrose l’argument principal pour l’arthroplastie d’emblée post traumatique sur les fractures à 3 ou 4 fragments [15]. La prothèse céphalique ne semble pas offrir de meilleurs résultats fonctionnels post opératoires par rapport à l’ostéosynthèse simple [16, 17]. Effectivement les résultats de l’hemiarthroplastie dépendent de la consolidation des tubérosités et nécessite une période de six semaine d’immobilisation, donc on se retrouve en fin de compte avec une épaule enraidie. L’avènement de la prothèse totale inversée vient doucement corriger ses défaillances avec une régression plus rapide de la douleur ainsi qu’une mobilité plus importante. Les fractures céphalotuberositaire à 3 et 4 fragments pourraient bénéficier d’une prothèse totale inversée minimisant le risque de reprise chirurgicale secondaire tout en permettant une autonomie confortable aux sujets âgés [18, 19].

## Conclusion

Il n y a pas une seule technique chirurgicale pour la prise en charge des fractures proximales complexes de l’humérus. L’enclouage centromédullaire permet une synthèse osseuse simple et au prix d’un abord limité avec des résultats fonctionnels très prometteurs. La comminution fracturaire et l’ostéoporose peuvent limiter ces indications. La synthèse par plaque vissée peut être un choix judicieux surtout pour le sujet jeune. Cependant le sujet âgé de plus de 70 peut bénéficier d’une arthroplastie totale inversée qui permet une autonomie confortable et indispensable à cet âge.

### Etat des connaissances actuelle sur le sujet

- Les fractures proximales de l’humérus sont très fréquentes représentant 5% de toutes les fractures;
- Leur répartition est bimodale touchant préférentiellement le sujet âgé ostéoporotique;
- Elles sont caractérisées par les difficultés de leur traitement, l’absence de technique de référence et certaines controverses: lesquelles opérées? Quelle technique pratiquée: prothèse? ostéosynthèse a ciel ouvert? ou percutanée?

### Contribution de notre étude à la connaissance

- L’enclouage centromédullaire permet une synthèse osseuse simple et au prix d’un abord limité avec des résultats fonctionnels très prometteurs;
- Il s’agit d’une technique peu couteuse et reproductible avec une courte courbe d’apprentissage.
